# Rod photoreceptor clearance due to misfolded rhodopsin is linked to a DAMP-immune checkpoint switch

**DOI:** 10.1074/jbc.RA120.016053

**Published:** 2020-11-27

**Authors:** Sang Joon Lee, Wei Wang, Lei Jin, Xiaoqin Lu, Lei Gao, Yao Chen, Tingting Liu, Douglas Emery, Eric Vukmanic, Yongqing Liu, Henry J. Kaplan, Douglas C. Dean

**Affiliations:** 1 Department of Ophthalmology and Visual Sciences, University of Louisville Health Sciences Center, Louisville, Kentucky, USA; 2 Department of Ophthalmology, Kosin University College of Medicine, Seo-gu, Busan, Korea; 3 Department of Ophthalmology, The Third People's Hospital of Dalian, Dalian Medical University, Dalian, China; 4 Department of Medicine, University of Louisville Health Sciences Center, Louisville, Kentucky, USA; 5 Department of Hematology, Xinqiao Hospital, Third Military Medical University, Chongqing, China; 6 Department of Ophthalmology, Xiangya Hospital, Central South University, Changsha, China

**Keywords:** retinal degeneration, retina, photoreceptor, retinal metabolism, myeloid cell, BSA, bovine serum albumin, CALR, calreticulin, DAMP, danger-associated molecular pattern, ER, endoplasmic reticulum, IS, inner segments, OCT, optimal cutting temperature, OS, outer segments, ONL, outer nuclear layer, PBS, phosphate buffered saline, PrCR, programmed cell removal, RP, retinitis pigmentosa, RHO, rhodopsin, SIRPa, signal regulatory protein alpha, WT, wild-type

## Abstract

Chronic endoplasmic reticulum stress resulting from misfolding of the visual pigment rhodopsin (RHO) can lead to loss of rod photoreceptors, which initiates retinitis pigmentosa, characterized initially by diminished nighttime and peripheral vision. Cone photoreceptors depend on rods for glucose transport, which the neurons use for assembly of visual pigment-rich structures; as such, loss of rods also leads to a secondary loss of cone function, diminishing high-resolution color vision utilized for tasks including reading, driving, and facial recognition. If dysfunctional rods could be maintained to continue to serve this secondary cone preservation function, it might benefit patients with retinitis pigmentosa, but the mechanisms by which rods are removed are not fully established. Using pigs expressing mutant RHO, we find that induction of a danger-associated molecular pattern (DAMP) “eat me” signal on the surface of mutant rods is correlated with targeting the live cells for (PrCR) by retinal myeloid cells. Glucocorticoid therapy leads to replacement of this DAMP with a “don't eat me” immune checkpoint on the rod surface and inhibition of PrCR. Surviving rods then continue to promote glucose transport to cones, maintaining their viability.

Protein synthesis can outpace protein folding in rapidly dividing cells, leading to accumulation of unfolded proteins in the endoplasmic reticulum (ER) (1, 2). An unfolded protein response (UPR) pathway is activated upon the resulting ER stress to promote folding and enhance cell viability (3, 4). Mutations that inhibit protein folding and transport out of the ER also lead to chronic stress. The rod photoreceptor visual pigment protein rhodopsin (RHO) can be mutated at multiple sites, and some of these mutations cause retention of unfolded protein in the ER, leading to stress (5). Rods do not tolerate this chronic ER stress, and their progressive loss causes retinitis pigmentosa (RP), leading to diminished nighttime and peripheral vision (5, 6). Although cone photoreceptors do not express RHO, they are dependent upon rods for glucose transport from the circulation, which they—like other neurons—require for viability (7, 8, 9, 10, 11, 12). Therapeutic approaches aimed at preventing rod loss and in turn cone loss in *RHO* mutation-initiated RP have utilized neuroprotective compounds and more recently attempts with gene editing to restore a wild-type (WT) sequence in mutant RHO (5, 13, 14, 15, 16, 17). But, neuroprotective strategies to date have failed to prevent RP progression.

ER stress induces expression of danger-associated molecular pattern (DAMP) proteins that serve as “eat me” signals on the cell surface for their recognition by macrophages and microglia (1, 18, 19, 20). One such DAMP is calreticulin (CALR), which translocates from the ER to the cell surface in response to ER stress (1, 21, 22, 23, 24). Resulting immune-mediated programmed cell removal (PrCR) then targets live cells with ER stress (2, 21). Despite this chronic stress and DAMP expression, cells frequently remain viable. A reason for this survival in the face of chronic ER stress and DAMP expression is resulting UPR induces expression of “don't eat me” proteins on their cell surface that serve as immune checkpoints to block T cell activation and macrophage/microglia phagocytosis, thereby dominantly preventing PrCR (21, 25, 26, 27, 28). These immune checkpoints include CD47, which binds to Sirpa on macrophage/microglia to signal inhibition of their phagocytosis pathway (25, 28, 29). Indeed, expression of CD47 is linked to UPR (4, 30).

These findings linking ER stress to PrCR raise the question as to whether PrCR might contribute to rod loss in RP patients with *RHO* mutations. Resident microglia in the retina (31) have been proposed to contribute to rod damage and loss in RP (32, 33), but other studies point to a beneficial role for the cells in survival of RPE and rods following light damage, at least in part, by removing dying cells and cell debris (34, 35, 36, 37). However, beyond resident microglia, monocytes are recruited to the retina during RP progression (37, 38), and they can mediate cell death following retinal detachment (39). Thus, populations of resident and recruited myeloid cells might have distinct functions in RP. We provide evidence that live rods undergo PrCR by retinal myeloid cells in RP, and this is a major cause of rod loss as RP progresses in pigs. This PrCR is associated with expression of chemotactic and inflammatory cytokines linked to migration of myeloid cells into the outer nuclear layer (ONL) of photoreceptors and to the DAMP, CALR, which acts as a myeloid recognition signal on the rod cell surface.

One neuroprotective factor is BDNF, and binding to its receptor, TrkB, activates the survival-promoting arm of UPR to enhance protein folding and viability (3, 40). A key response to ER stress is phosphorylation and inactivation of eIF2a, leading to changes in translation of proteins mediating ER stress (41). This phosphorylation and inactivation of eIF2a appear sufficient to cause relocalization of CALR to the cell surface (42). TrkB signaling causes induction of ATF4, leading in turn to induction of Gadd34 (41). Notably, Gadd34 in complex with PP1 dephosphorylates eIF2a to de-escalate the stress response and thus restrict surface CALR (41). Particular interest has focused on therapeutic activation of the BDNF/TrkB pathway for neuroprotection in diseases involving entangled proteins that trigger ER stress including Huntington's, Alzheimer's, and *RHO* mutations in RP (43, 44, 45). BDNF-dependent activation of TrkB is transient and confined to the early response to ER stress (3). We show that TrkB is not activated on WT photoreceptors, nor it is active as mutant RHO chronically accumulates in the ER during rod loss in RP progression in pigs. Thus, we reasoned if TrkB could be activated, it might impact cell surface CALR and rod survival in RP. Long-term delivery of high levels of BDNF to activate TrkB in the brain and retina has been a challenge. However, TrkB can be activated by glucocorticoids, short-circuiting a requirement for BDNF (46, 47, 48). Although glucocorticoids have proven useful in treating macular edema associated with RP, they have not shown effectiveness in reversal of rod functional loss in patients (49); however, it is possible that early treatment with glucocorticoids to activate TrkB, prior to evidence of rod functional loss, might prevent or delay mutant rod loss in RP.

Here, we show that a single intravitreal injection of a slow release glucocorticoid can activate TrkB for more than 2 months on pig photoreceptors. Such therapy prevented surface CALR and rod loss during this period, but only when administered early in the disease process, prior to onset of rod degeneration. And, with mutant rod survival, we show that glucose transport to cones and cone outer segments (OS) synthesis are maintained in pig RP.

## Results

### Induction of chemotactic and myeloid activating cytokines in RP

Prior to birth, WT and mutant RHO are transported into membranous OS in rods that house the visual pigment in *RHO* mutant pigs (7, 11). Light causes a conformational change in the RHO N-terminal domain that couples with transducin for signal transduction (5). We observed initiation of RHO accumulation in the ER of photoreceptor inner segments (IS) shortly after light exposure at birth, suggesting a light-induced conformational change is initiating retention of misfolded P23H RHO in the ER of pig rods, and *via* aggregation with misfolded mutant RHO, WT RHO is also retained in the ER (7). Misfolded RHO is ultimately exported from the ER to the cytoplasm for ubiquitin-mediated degradation (5). Accumulation of RHO in the ER causes ER stress, which in turn activates UPR in an attempt to promote protein folding and survival (3). But, we observed rod loss by P30 in pig RP (Fig. S1) (7), demonstrating that UPR activation is not preventing rod loss in this model. As opposed to rods, cones survive in RP pigs (Fig. S3), but with rod loss, glucose transport into cones is diminished and their OS are lost (7, 11). Like other neurons, cones are dependent upon glucose, which they utilize for energy and synthesis of new OS tips, following their daily phagocytosis by adjacent retinal pigment epithelium (7, 8, 9, 10, 11, 14). With rod loss, cones begin to lose OS at P30, and all OS are lost by P90 (7).

**Figure 1:**
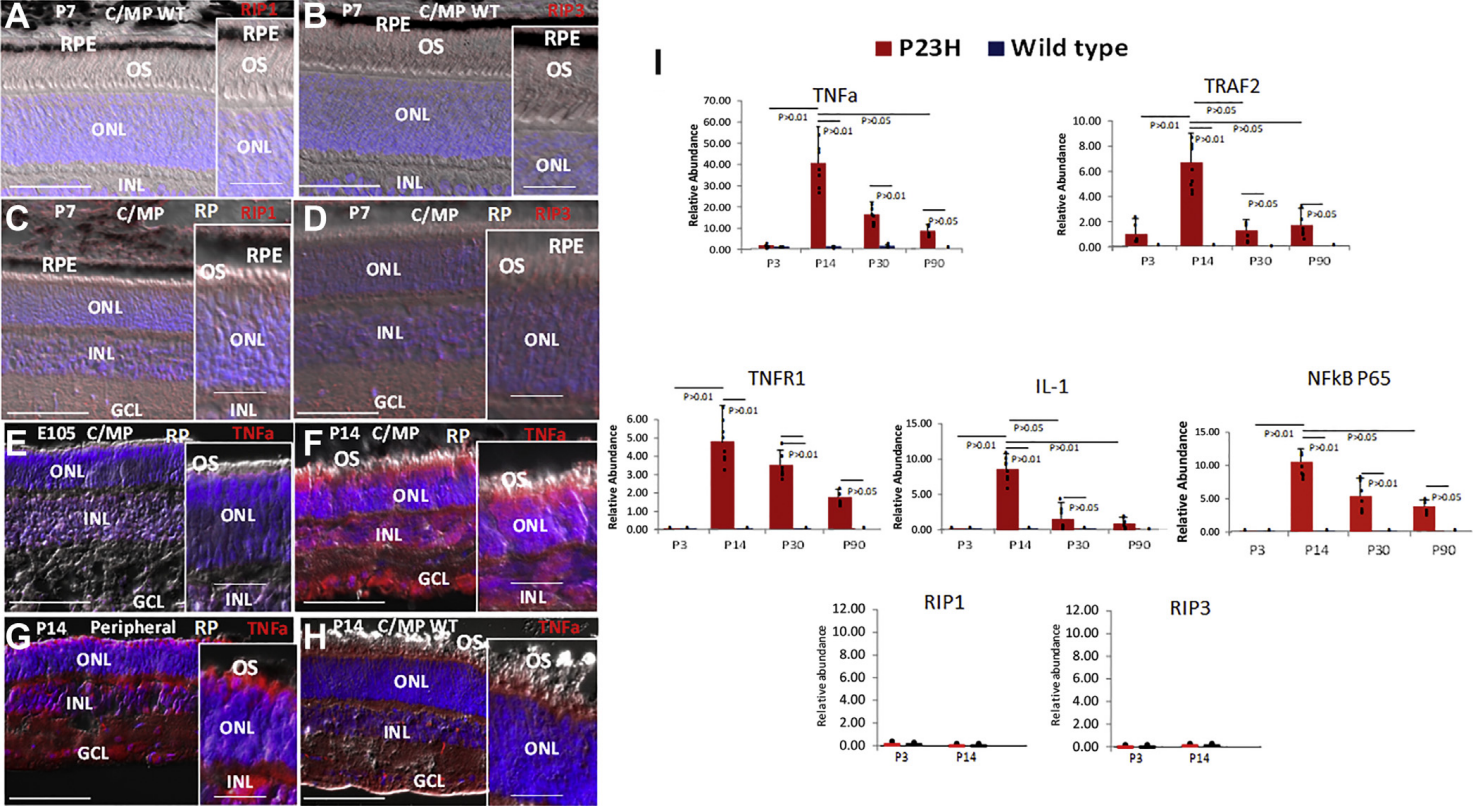
**TNFa and NFKB pathway activation in pig RP is not linked to expression of RIP1 and RIP3 necrotic drivers.***A–D*, RIP1 and RIP3 are not evident in WT or RP pig retinas. *E*, representative immunostaining showing no detectable TNFa expression in the retina of RP pigs at E105 before birth. *F* and *G*, immunostaining showing TNFa expression in representative regions of the central/mid-peripheral (C/MP) (sections 19–23 in Fig. S1*C*) and peripheral (sections 3–7, 35–39) retinas of RP pigs at P14. *H*, lack of TNFa expression postnatally in the WT pig retina. Bars are 50 μm (10 μm in the panel *insets*). *I*, real-time PCR of isolated retinas was used to compare expression of mRNAs in the TNFa and NFKB pathways during pig RP (P23H) progression to WT retinas. Bars are averages and error bars are standard deviations. Replicates of three samples each from three different retinas are shown. GCL, ganglion cell layer; INL, inner nuclear layer; ONL, outer nuclear layer; OS, outer segments; RPE, retina pigment epithelium.

**Figure 2:**
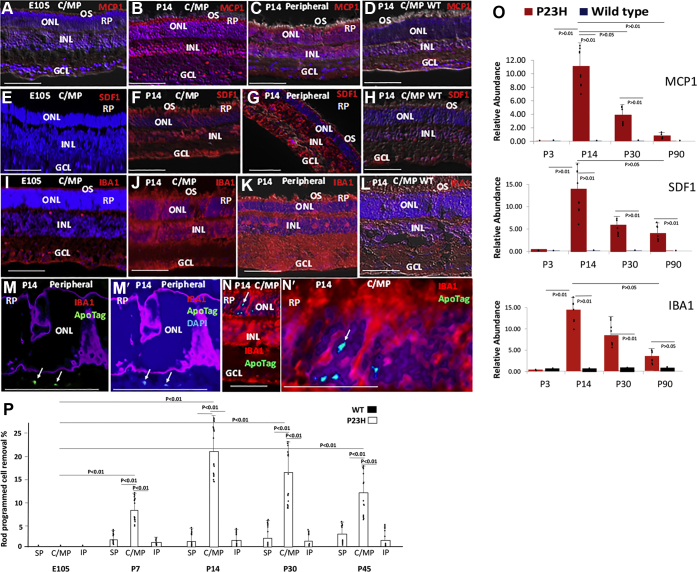
**Expression of myeloid cell chemotactic cytokines, and PrCR of rods by IBA+ myeloid cells in pig RP.***A–C*, immunostaining showing expression of MCP1 prior to birth at E105 and after birth at P14 in central/mid-peripheral (C/MP) and peripheral regions of RP pig retinas (see Figs. 1 and S1). *D*, lack of MCP1 in the WT pig retina. *E–G*, lack of SDF1 prior to birth at E105, but induction after birth at P14 in central/mid-peripheral (C/MP) and peripheral regions of RP pig retinas. *H*, lack of SDF1 in the WT pig retina. *I–K*, expression of IBA1 prior to birth at E105 and after birth at P14 in C/MP and peripheral regions of RP pig retinas. *L*, reduced IBA1 expression in WT pig retina compared with RP retinas. *M–N*ʹ, immunostaining showing IBA1+ myeloid cells surrounding photoreceptors in the ONL of RP pigs. Note that apoptotic cells (*arrows*) (*green* in the absence of DAPI in panel *M*, and *cyan* when DAPI is included in panel *Mʹ*) are not being phagocytosed by IBA1+ cells. Bars are 50 μM. *O*, real-time PCR of isolated retinas was used to compare expression of mRNAs for MCP1, SDF1, and IBA1 during pig RP progression to WT retinas. *P*, DAPI+ rod nuclei surrounded by IBA1+ cells were counted as cells undergoing PrCR in the central/mid-peripheral (C/MP) (sections 19–23 in Fig. S1*C*), superior peripheral (SP) (sections 35–39), and inferior peripheral (IF) (sections 3–7) of WT and RP pig retinas beginning before birth at E105 and ending at P45. The percentage of cells in the ONL undergoing PrCR is shown. Three high-power views (∼800 μM in length) were counted for each section. Bars are averages and error bars are standard deviations. Results are representative of three different retinas. GCL, ganglion cell layer; INL, inner nuclear layer; ONL, outer nuclear layer; OS, outer segments.

We followed photoreceptor apoptosis during the time course of rod loss in the pigs (Fig. S1). As in other RP models, rod apoptosis was evident regionally in the pig retina, but it did not appear to account for all rod loss during RP progression. Notably, similar rod and cone apoptosis was evident at P7, but cone number did not diminish during RP progression (7). RIP1 and RIP3 mediate necrosis in response to TNFa and NFkB (50). As shown previously in mouse RP, neither RIP1 nor RIP3 was evident during the period of rod loss (51) (Fig. 1, *A*–*D*). But, we observed TNFa/NFkB pathway activation in the ONL comprising photoreceptor cell bodies in pig RP (Fig. 1, *E*–*I*). Beyond their ability to initiate RIP-dependent necrosis under some conditions, TNFa and NFkB are inflammatory cytokines that activate macrophages/microglia to promote their phagocytic pathway (52). And, in addition to TNFa and NFkB, we also observed induction of MCP1 and SDF1 in the ONL (Fig. 2, *A*–*H* and *O*), which are chemotactic cytokines that promote migration of macrophages/microglia (53). We then followed macrophages/microglia during pig RP progression by immunostaining for the myeloid marker IBA1 and found that after birth myeloid cells migrated into the ONL and sent projections into the ONL surrounding and apparent engulfing rods in RP pigs (Fig. 2, *I*–*O*) (Fig. 2, *M–Nʹ* and *P*). Notably, rods positive for apoptosis were not being surrounded by these myeloid cells (Fig. 2, *M–Nʹ*). We concluded that, as with cancer cells, live rods are being targeted for PrCR.

### CALR translocates from the ER to the surface of mutant rods

**Figure 3:**
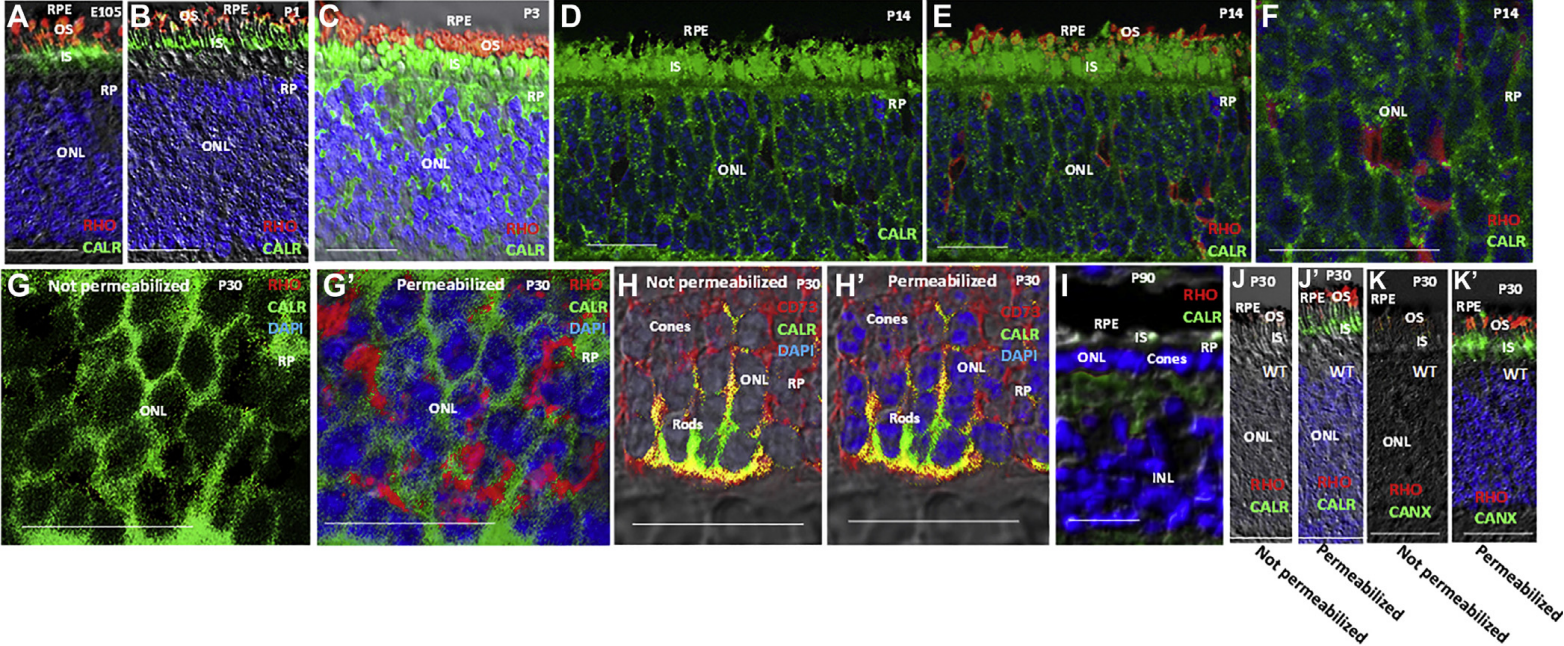
**Translocation of CALR from the ER of inner segments (IS) to the rod cell surface in RP pigs.***A–F*, immunostaining showing that CALR is retained in the ER of IS before birth and at P1 in RP pig retinas, but it translocates from the IS to cell bodies in the ONL by P3 and is maintained in this location at P14. Note, at E105 and P1, RHO is in OS, but by P14 it is retained in IS, and at this age, RHO is starting to be translocated to cells bodies in the ONL for degradation. There is little or no overlap between RHO and CALR in cell bodies at P14. *G–Gʹ*, by P30 in RP pig retina, CALR continues to be expressed on cell bodies in the ONL, and more RHO has moved to cell bodies. In panel *G*, the frozen section was not permeabilized with triton prior to immunostaining, and consistently DAPI nuclear staining was not evident. In panel G*ʹ*, the section was then permeabilized with triton, and DAPI nuclear staining is evident. Note, RHO immunostaining required cell permeabilization, indicating its cytoplasmic localization, whereas CALR staining is evident without permeabilization, demonstrating extracellular localization. *H–Hʹ*, as a control, CD73 is also expressed on the surface of photoreceptors in the retina of RP pigs. As in panels *G* and *Gʹ*, panel *H* shows no permeabilization and *Hʹ* after permeabilization. Cones comprise the outer two rows of the ONL in the pig retina, with rods comprising the inner rows. Note, expression of CALR and CD73 on the surface of rods in the ONL, but CD73 is also expressed on the surface of cones. *I*, by P90 in the RP pig retina, rods are lost and cones persist as a single row in the ONL, where they maintain IS but not OS and show no surface expression of CALR. *J–Jʹ*, CALR is retained in the ER of inner segments (IS) and RHO in OS at P30 in WT pigs. The section was photographed before and after triton permeabilization as in panels *H* and *Hʹ*. *K–Kʹ*, As with CALR, A second ER marker, CANX, is also retained in the ER of IS at P30 in WT pigs. The section was again photographed before and after permeabilization. Bars are 50 μM. Immunostaining is in the C/MP region (see Figs. 1 and S1). Representative immunostaining of three different retinas is shown. INL, inner nuclear layer; ONL, outer nuclear layer; OS, outer segments; RPE, retina pigment epithelium.

Upon ER stress, the ER protein CALR can translocate to the cell surface, where it serves as a DAMP recognition signal for macrophages/microglia (1, 21, 22, 23, 24). We found that CALR translocated from the ER to the rod cell body following birth in RP pigs (Fig. 3, *A*–*F*). We coimmunostained with the photoreceptor cell surface marker CD73 (54) and found that CALR appeared with CD73 on what appeared to be the surface of rods, which constitute the inner rows of the ONL (Fig. 3, *H* and *Hʹ*). To verify surface expression, tissue frozen sections were immunostained prior to and following cell permeabilization, which we monitored by DAPI nuclear staining. We found that CALR and CD73, but not RHO, immunostaining was evident without cell permeabilization, demonstrating surface expression of CALR on rods (Fig. 3, *G*–*Hʹ*). During this period, RHO trapped in the ER was beginning to be transported to the cytoplasm for degradation, and notably this cytoplasmic RHO did not overlap with cell surface CALR in coimmunostaining experiments (Fig. 3, *G* and *Gʹ*). Together, these results demonstrate translocation of CALR from the ER to the surface of rods after birth in RP pigs. By P90, rods were lost and cones lacking OS but maintaining IS persisted, and CALR was not associated with their cell bodies (Fig. 3*I*). As a control, WT retinal sections were immunostained for RHO and CALR or another ER marker CANX prior to permeabilization, and then the sections were permeabilized (Fig. 3, *J*–*Kʹ*). The results show coexpression of CALR and CANX in the ER of IS of WT retinas.

### Glucocorticoids activate TrkB on rods in the ONL

**Figure 4:**
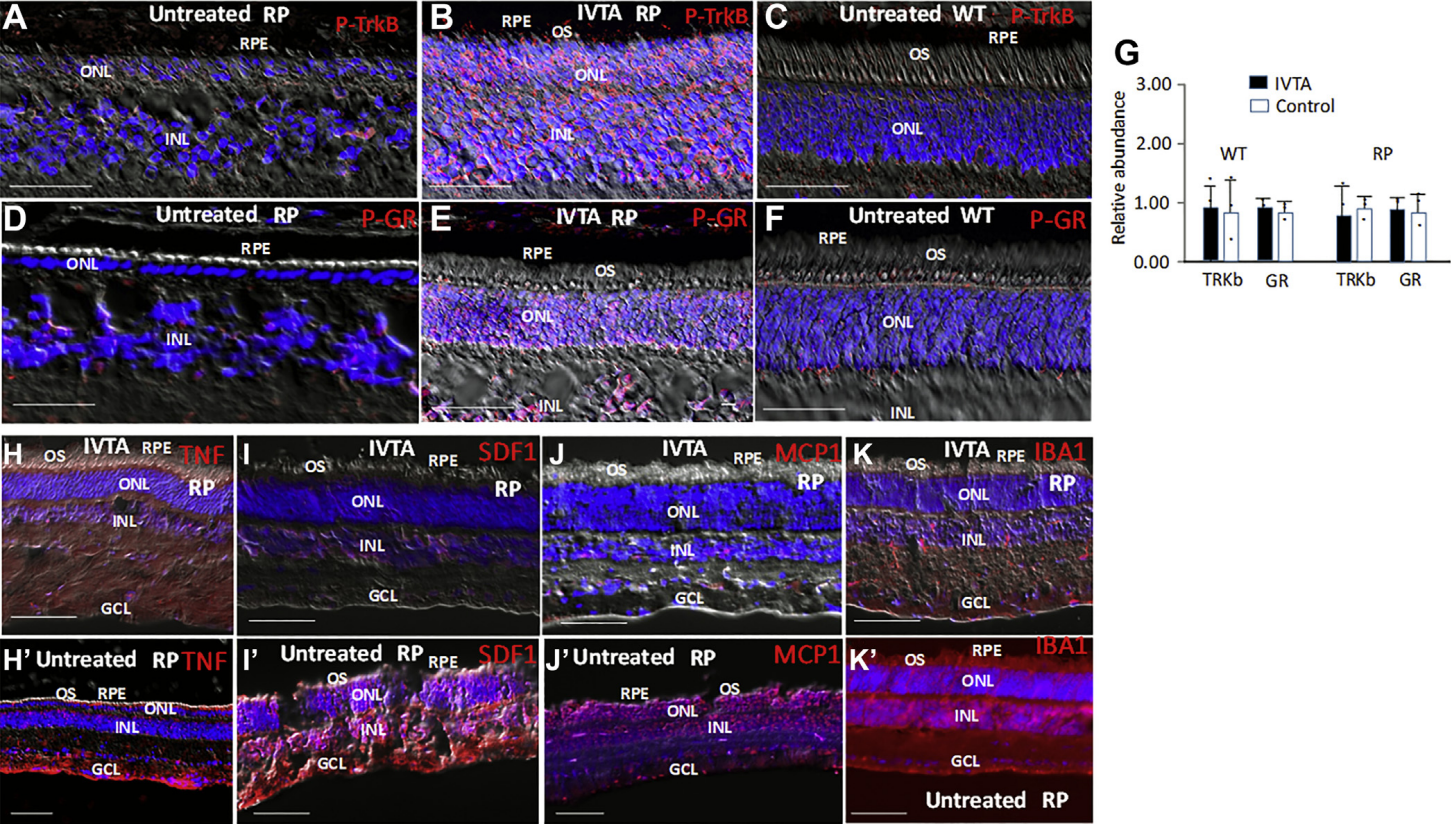
**IVTA injection causes activating phosphorylation of the glucocorticoid receptor (P-GR) and the TrkB receptor (P-TrkB), inhibits expression of TNFa, MCP1 and SDF1 and recruitment of IBA1+ cells to the ONL of RP pig retinas.** RP or WT pigs were injected with IVTA or a sham control (untreated) at P7, and immunostaining was at P65. *A–C*, P-TrkB is not expressed in the retinas of WT or RP pigs following sham injection, but it is induced following IVTA injection. *D–F*, phosphorylated and activated GR (P-GR) is not expressed in the retinas of WT or RP pigs following sham injection, but it is induced in the RP pig retina following IVTA injection. Note there is an increase in ONL rows following IVTA treatment of RP pigs (*E*) compared with the sham control in *D*. *G*, real-time PCR showing IVTA or sham injection does not affect expression of TrkB or GR mRNA levels in WT or RP pig retinas. Results were normalized to ACTB mRNA. Three retinas are averaged, and error bars are standard deviations. *H–Kʹ*, immunostaining showing that expression of TNFa, MCP1, and SDF1 is reduced in the RP pig retina along with recruitment of IBA1+ cells to the ONL following IVTA injection compared with sham-injected controls. Representative immunostaining of three different retinas is shown. Bars are 50 μm.

Binding of BDNF to its cell surface receptor, TrkB, triggers activating phosphorylation of TrkB (55, 56, 57, 58). TrkB in turn activates the prosurvival arm of UPR, which is directed at promoting protein folding and cell survival in response to ER stress (5, 40). But, this arm of UPR is only activated transiently following ER stress, and its loss of activation in chronic ER stress is linked to onset of cell loss (3, 59). We found that TrkB was not phosphorylated/activated in the retina in WT pigs or during the period of rod loss in RP pigs (Fig. 4, *A*–*B*), raising the possibility that its therapeutic activation might serve to diminish mutant rod loss.

Interestingly, glucocorticoids trigger activating phosphorylation of TrkB in a BDNF-independent fashion (46, 47, 48), and blocking glucocorticoid action was shown to cause photoreceptor loss (60). Intravitreal injection of the slow release glucocorticoid triamcinolone is widely used in patients to treat conditions such as macular edema (61, 62). Pharmacodynamics indicate continuous release for more than 1 month following injection (62, 63). We injected triamcinolone intravitreally (IVTA) into RP and WT pigs at P7 and examined glucocorticoid receptor activating phosphorylation at P14. As anticipated, we found that early IVTA at P7 induced glucocorticoid receptor phosphorylation in both RP and WT pig photoreceptors, and this was maintained at P30 (Fig. 4). We then examined activating phosphorylation of TrkB and found that IVTA injection also activated TrkB in both WT and RP photoreceptors, which was maintained at P30 (Fig. 4*F*). As a control, IVTA did not affect expression of GR or TrkB mRNA levels in WT or RP pig retinas (Fig. 4*G*). We concluded that IVTA was activating both the glucocorticoid receptor and TrkB in the ONL of WT and RP pigs.

### IVTA inhibits rod PrCR

**Figure 5:**
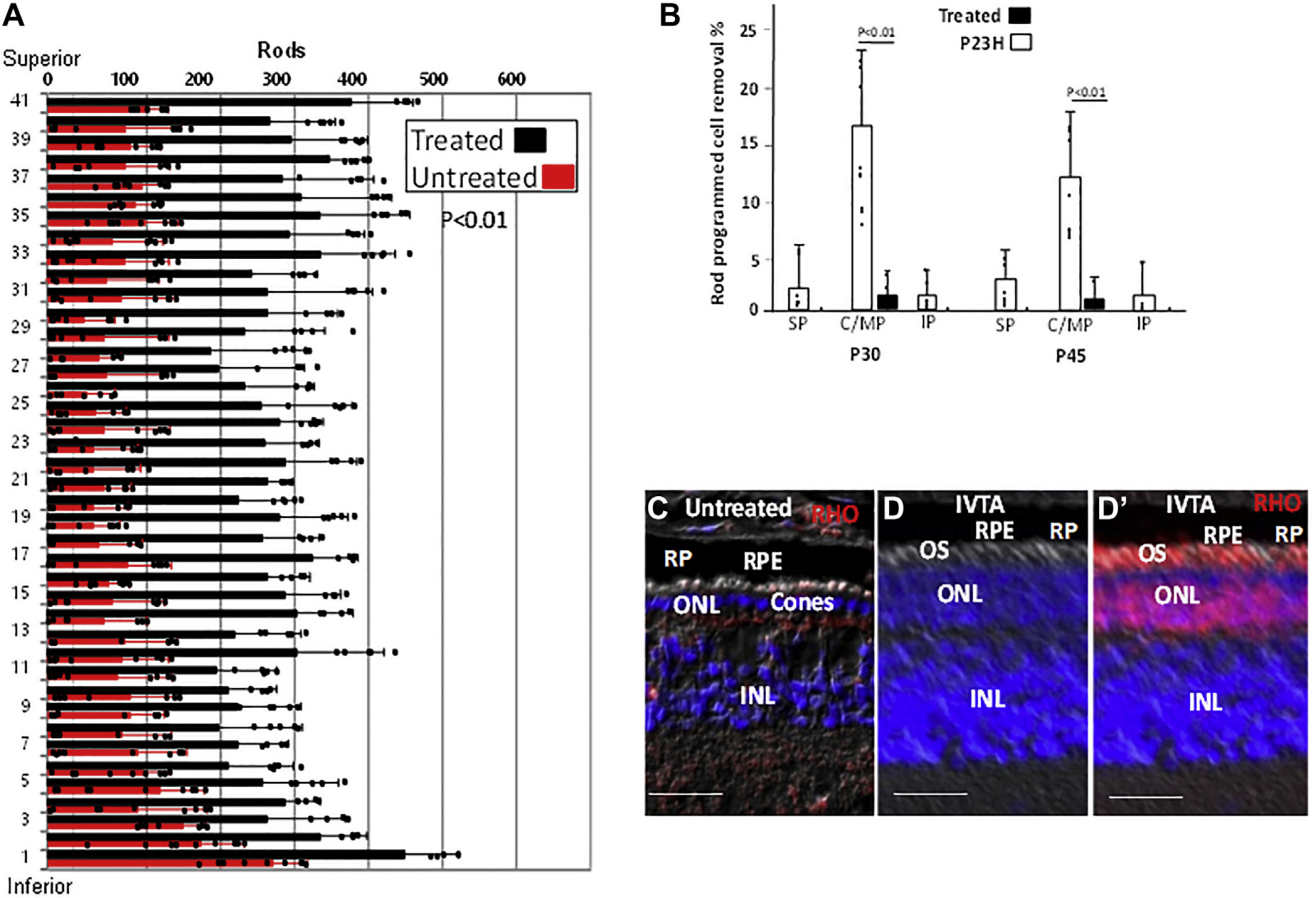
**IVTA injection inhibits rod PrCR in RP pig retinas.***A*, rods were identified and quantified as in Figure S1 at P45 following IVTA or sham injection at P7 in RP pigs. Bars are averages and error bars are standard deviations. Three high-power views were counted for each section in three different retinas. *B*, PrCR by IBA1+ cells was quantified in RP pig retinas as in Figure 4 following IVTA (treated) or sham injection (P23H) at P7. Bars are averages and error bars are standard deviations. Results are representative of three different retinas. *C*, immunostaining showing loss of RHO+ rods at P65 following sham injection at P7. *D–Dʹ*, immunostaining showing retention of ONL rows and movement of RHO (panel *Dʹ*) into OS at P65 following IVTA injection at P7. Bars are 50 μm. Immunostaining is in the C/MP region (see Fig. S1). INL, inner nuclear layer; ONL, outer nuclear layer; OS, outer segments; RPE, retina pigment epithelium.

Next, we examined the effect of IVTA on expression of chemotactic and inflammatory cytokines. Following IVTA injection at P7, MCP1, SDF1, and TNFa were diminished in the ONL of RP pigs compared with sham-injected controls at P30 (Fig. 4, *G*–*I*). Consistently, migration of IBA1+ myeloid cells into the ONL was reduced following IVTA (Fig. 4*J*), leading to diminished rod PrCR and a corresponding increase in rod number in the ONL at P45 (Fig. 5, *A* and *B*).

### RHO diminishes in the ER following IVTA injection in RP pigs

Activation of UPR to promote protein folding has been shown to reduce aggregation of P23H mutant RHO with WT RHO, promoting movement of the WT protein to OS while the mutant protein is shuttled to the cytoplasm for degradation (5). Because TrkB is inactive in the ONL during pig RP progression, we followed RHO localization after injection of IVTA to activate TrkB. Following IVTA injection, RHO accumulating in the ER of rod IS was reduced, and two pools of RHO were evident, one in the cytoplasm and the other in OS (Fig. 5, *C*–*Dʹ* and Fig. S2). Although we cannot discriminate between WT and P23H mutant RHO by immunostaining *in vivo*, it is likely based on previous findings that IVTA activation of TrkB is reducing protein aggregation, allowing mutant RHO in the ER to be transported to the cytoplasm for degradation, and WT RHO, previously retained in the ER *via* aggregation with mutant RHO, to be transported into OS. Consistent with cytoplasmic RHO representing P23H mutant protein undergoing degradation, all cytoplasmic RHO lacked the N-terminal domain (Fig. S2), which is the site of the P23H mutation, and such N-terminal cleavage of P23H RHO has been reported previously (5, 64). As noted above, with this diminished accumulation of RHO in the ER following IVTA injection, mutant rod survival was increased (Fig. 5*A*).

### IVTA suppresses cell surface CALR on mutant rods

**Figure 6:**
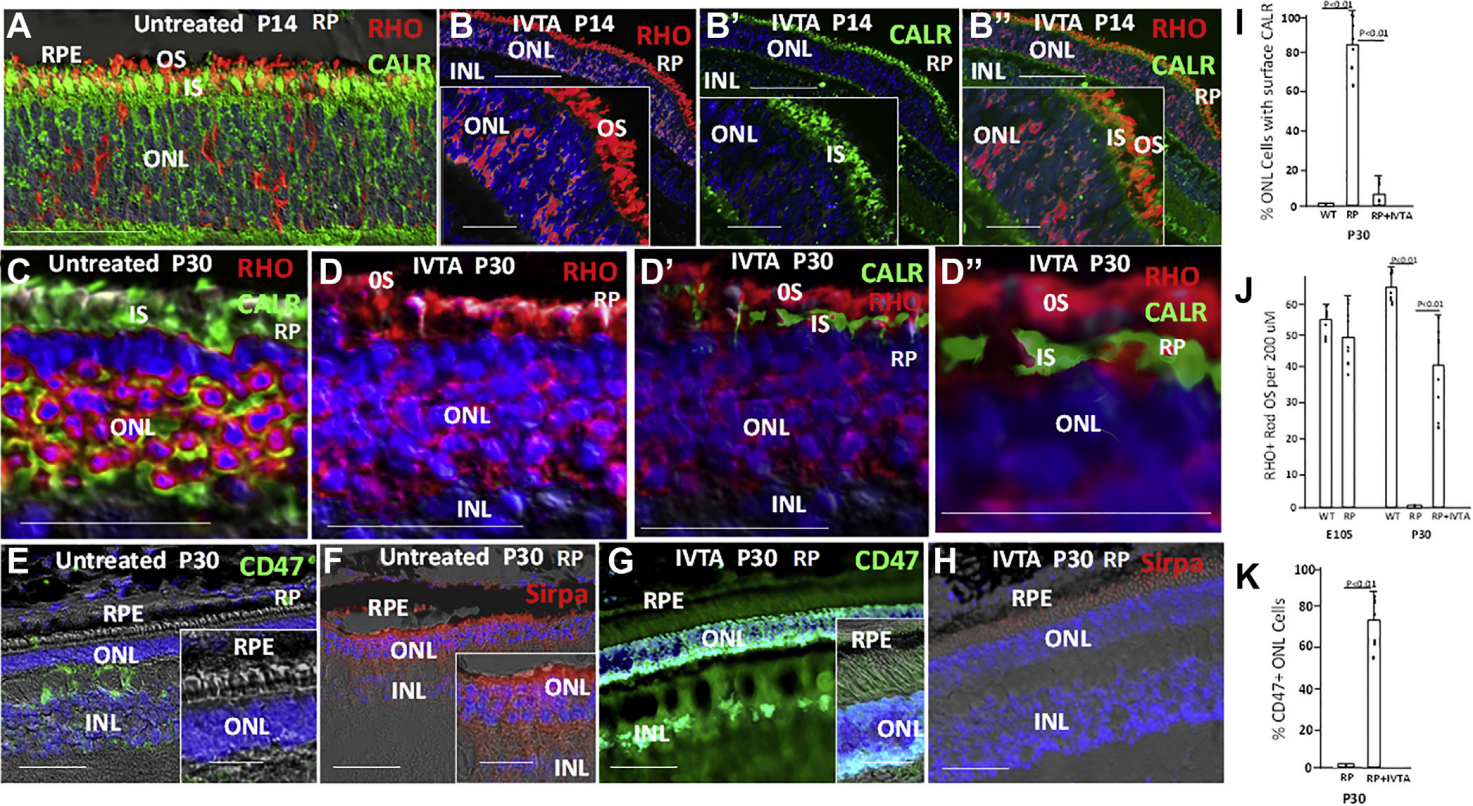
**IVTA injection prevents CALR translocation to the rod cell surface, it induces CD47 on rods, and it inhibits recruitment of SIRPa+ cells to the ONL in retinas of RP pigs.***A*, immunostaining of pig RP retina at P14 following sham injection at P7. Note retention of RHO in the ER of IS and onset of its translocation to cell bodies in the ONL. At this point, CALR is moving from the IS to cell bodies in the ONL. *B–B″*, IVTA injection at P7 prevents CALR movement from the ER in IS to cell bodies at P14. Little RHO is evident in IS, instead there are two populations of RHO, one in cell bodies, and there other in OS. *C–D″*, as at P14, IVTA injection at P7 maintains CALR in the ER of IS, and it allows RHO to translocate into OS at P30. *E*, CD47 is not expressed on photoreceptors in the ONL of RP pig retinas at P30 following sham injection at P7. *F*, SIRPa+ cells are recruited to the ONL of RP pig retinas at P30 following sham injection at P7. *G*, IVTA injection at P7 results in CD47 expression in the ONL of RP pig retinas at P30, primarily on rods in inner rows of the ONL. *H*, IVTA injection at P7 results in diminished recruitment of SIRPa+ cells to the ONL of RP pig retinas at P30. Immunostaining is representative of three different retinas. Bars are 50 μm (10 μm in panel *insets*). *I*, cell surface CALR was identified by cell permeabilization as in Figure 3. Three high-power views of ∼800 μm each in three differentia retinas were counted. *J*, RHO+ OS were counted in 200 μm regions in three retinas as in Figure S3. *K*, CD47 immunostaining of ONL cells was counted in three high-power views of ∼800 μm each in three differentia retinas. Bars are averages and error bars are standard deviations.

PrCR of live cancer cells has been shown to be dependent upon at least three factors: (1) chemotactic migration of macrophages/microglia cells toward the cancer cells; (2) expression of a DAMP, such as CALR, on the cell surface, which serves as a recognition signal for macrophages/microglia; (3) expression of immune checkpoints on cancer cells that block macrophage/microglia phagocytic activity, thereby preventing PrCR. Above, we show that IVTA injection inhibits expression of chemotactic and inflammatory cytokines in the ONL and migration of microglia toward rods, thereby targeting the first factor in PrCR (Fig. 4, *G*–*J*). We then followed expression of CALR after IVTA injection and found that after IVTA at P7, CALR was retained in the ER of IS at P14 (Fig. 6, *A*–*B″* and *I*). And, as shown above (Fig. 5, *C*–*Dʹ*), RHO at P14 was reduced in the ER and pools of the protein were evident in OS and in the cytoplasm (Fig. 6, *A*–*B″* and *J*). By P30, much of the RHO could be found in cell bodies in the ONL in RP pigs, and CALR was still evident on the surface of cells in the ONL (Fig. 6*C*). As at P14, CALR continued to be retained in the ER at P30 following IVTA at P7 (Fig. 6, *D*–*D″* and *I*). And, RHO continued to be reduced in the CALR+ ER in IS, and two pools in cell bodies in the ONL and in OS were still evident (Fig. 6, *D*–*D″* and *J*). We concluded that IVTA is blocking transport of CALR from the ER to the surface of mutant rods, likely through activation of TrkB. Thus, IVTA is also targeting the second factor regulating PrCR.

### IVTA induces an immune checkpoint on mutant rods

CD47 on cancer cells interacts with signal regulatory protein alpha (SIRPa) on macrophages/microglia to signal a block in their phagocytosis. Blocking the CD47–SIRPa interaction then promotes phagocytosis and PrCR (26, 28, 65, 66, 67), and in atherosclerosis it enhances phagocytic removal of diseased vascular cells and halts disease progression (68). CD47 is not expressed on mature photoreceptors in mice (54), and we did not detect its expression on photoreceptors in either WT or RP pigs (Fig. 6, *E*–*K* and Fig. S5). However, CD47 was induced on rods at P30 following IVTA injection at P7 in both RP and WT pigs (Fig. 6, *G*–*K* and Fig. S5). These findings demonstrate that IVTA, likely through activation of TrkB, establishes a CD47 immune checkpoint on mutant rods. Consistent with IVTA inhibition of IBA1+ macrophage/microglia migration observed above (Fig. 4*J*), we found that immunostaining for the CD47 ligand, SIRPa, on macrophage/microglia was likewise diminished, providing further evidence that IVTA was inhibiting myeloid cell migration to the ONL in RP pigs (Fig. 6, *F*–*H*).

### IVTA-mediated rod survival maintains cone glucose transport and cone OS synthesis

**Figure 7:**
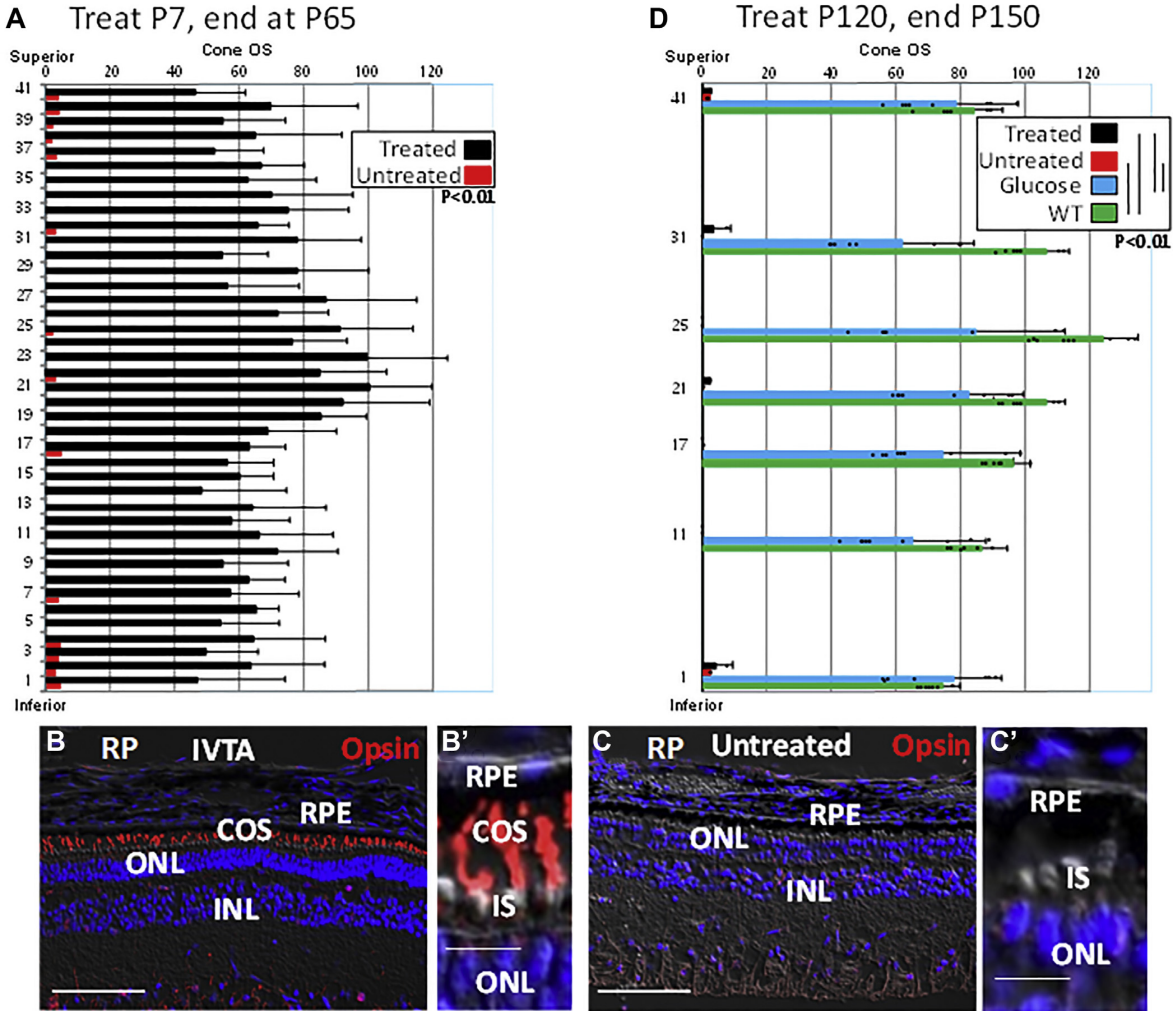
**Preservation of rods allows IVTA to maintain cone OS in retinas of RP pigs.***A*, IVTA or sham injections were at P7 and retinas from PR pigs were analyzed at P65. Cones were identified as in Figure 1, and cone OS were quantified after immunostaining for cone opsin and counted as in Figures S1 and 5. Three high-power fields were counted for each section in three retinas. *B–Cʹ*, representative immunostaining for cone opsin in the C/MP retina showing identification of cone OS in IVTA and sham-injected RP pigs at P65. *D*, IVTA injection following rod loss at P120 does not restore cone OS at P150. Cone OS were quantified as in *A* and in Figures S1 and 5. WT controls are at P150. As a positive control for restoration of cone OS following rod loss, glucose was injected into the subretinal space at P147 and retinas were analyzed at P150, as we described previously (7). Three high-power fields were analyzed in three different retinas for each section. Bars are averages and error bars are standard deviations. Bars are 50 μm in panels *B* and *C* and 8 μm in panels *Bʹ* and *Cʹ*.

As with other neurons, photoreceptors depend upon glucose, which they use for energy and building blocks for their daily synthesis of new OS (7, 8, 9, 10, 11, 14). Rods are necessary for efficient transport of glucose to cones, and, in the absence of rods, glucose starvation in cones leads to their loss of OS synthesis (7, 8, 9). We followed cone OS after rod loss in pig RP and found that IVTA injection at P7 largely prevented loss of cone OS at P60 (Fig. 7, *A*–*Cʹ*). However, when IVTA was injected later at P120, after rod loss, cone OS were not restored at P150 (Fig. 7*D*). We then conclude that it is IVTA protection of rods that is responsible for maintaining cone OS.

**Figure 8:**
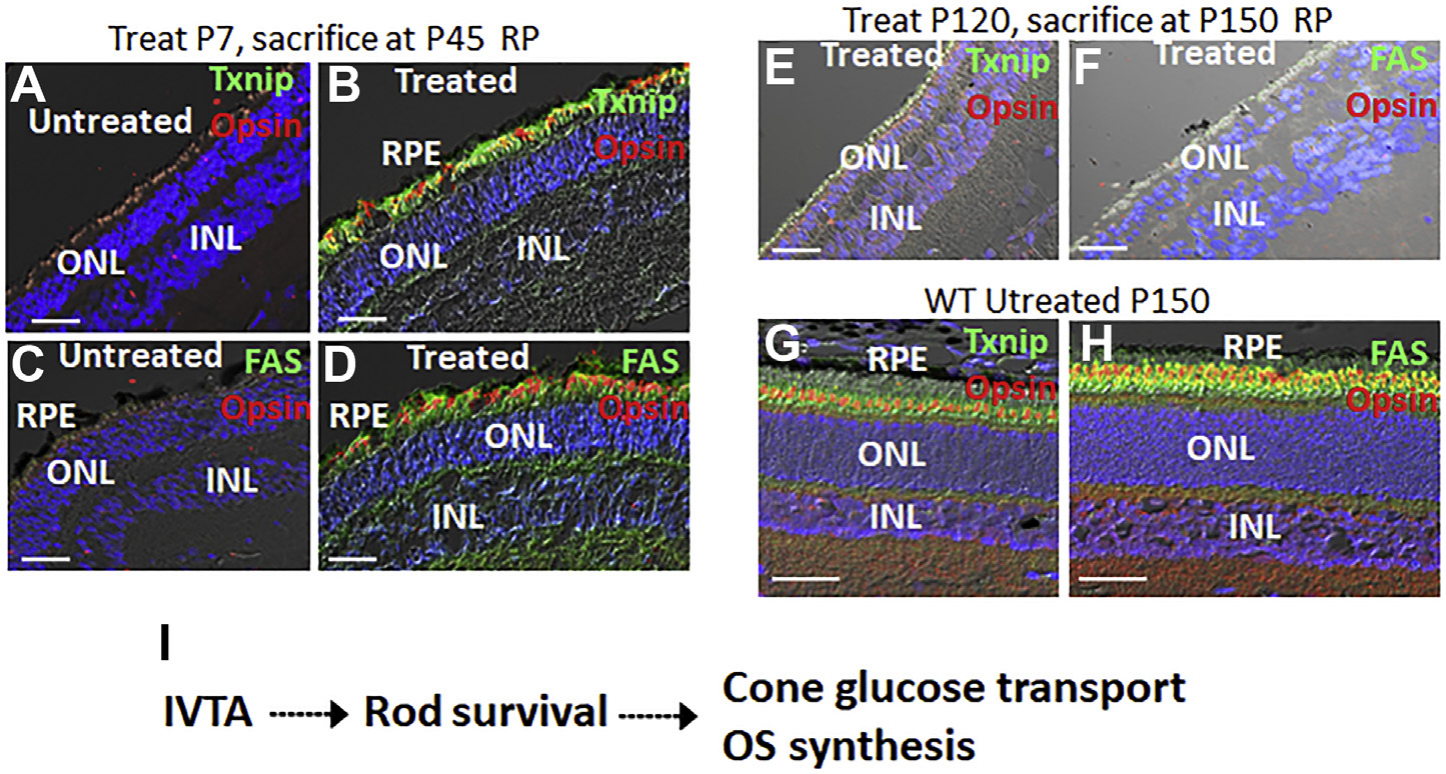
**IVTA injection maintains expression of glucose-dependent genes in the ONL of RP pigs.***A* and *B*, IVTA or sham injection was performed at P7, and retinas from RP pigs were analyzed at P45. Txnip expression is induced in IS along with cone opsin in OS. *C* and *D*, as with Txnip, FAS is also induced by IVTA. *E* and *F*, IVTA injection at P120, after rod loss, does not lead to increased expression of Txnip or FAS in the retina of RP pigs at P150. *G* and *H*, expression of Txnip and FAS in WT pig retinas at P150. Representative immunostaining of three separate retains is shown. Bars are 50 μm. *I*, model of IVTA therapy.

Emphasizing that glucose starvation in the absence of rods is responsible for cone OS loss, injection of glucose into the subretinal space of RP pigs at P147, after rods are lost, led to restoration of cone OS at P150 (Fig. 7*D*) (7). Tnxip is the most glucose-responsive gene identified, and expression of fatty acid synthase (FAS) is likewise glucose-dependent (7, 69). As we reported previously (7), expression of Txnip and FAS is diminished in the ONL of RP pigs (Fig. 8, *A*, *C*, *G*, and *H*). However, both Txnip and FAS were induced at P45 following IVTA injection at P7 in RP pigs compared with sham-injected controls (Fig. 8, *A*–*D*), suggesting that induction of these glucose-dependent genes is a marker of renewed glucose transport. Consistently, in mice and pigs expressing P23H Rho, we have shown using both labeled glucose and mass spectroscopy that glucose transport to cones is dependent upon rods in both pigs and mice (7, 11). In contrast to early injection, IVTA injection at P120, after rods were lost, failed to induce either Txnip or FAS at P150 (Fig. 8, *E*–*F*), suggesting that IVTA-dependent preservation of rods following early injection is responsible for maintaining glucose transport to cones for ongoing OS synthesis. Thus, once rod are lost, IVTA is no longer effective in restoring cone OS.

## Discussion

We provide evidence that PrCR of live rods contributes to loss of the photoreceptors in pig RP. Translocation of the DAMP, CALR, to the surface of rods with misfolded mutant RHO coincides with their PrCR by retinal myeloid cells (Fig. 8*I*). During this process, TrkB is not activated, indicating that the early BDNF/TrkB-inducible neuronal survival pathway is diminished with chronic ER stress at the point of rod loss in RP progression. Glucocorticoids bypass BDNF in activation of TrkB in photoreceptors, and slow release of glucocorticoids *via* a single IVTA injection can promote TrkB activation and rod survival for months. But, this therapy is only effective early in RP before onset of rod degeneration. We provide evidence that IVTA therapy has four consequences that influence mutant rod survival (Fig. 8*I*). First, it diminishes chemotactic and inflammatory cytokines, which attract and activate macrophages/microglia. Second, it maintains CALR in the ER, removing a DAMP from the surface of rods. Third, it induces cell surface expression of the immune checkpoint CD47, which binds SIRPa on the myeloid cell surface to inhibit their phagocytic pathway. Finally, IVTA leads to loss of RHO accumulation in the ER, with what we suggest is mutant RHO being translocated to the cytoplasm for degradation and WT RHO moving into OS. Notably, glucocorticoids are also classic inhibitors of the inflammatory response. Thus, is it likely that IVTA is functioning *via* a combined effect in RP.

Because rods are essential to maintain cone viability, we examined the effect of IVTA injection on cones. Rods are necessary for efficient glucose transport into cones, which like other neurons depend upon glucose, which they utilize for energy and as building blocks for new OS synthesis following daily removal of OS tips by the retinal pigment epithelium. We followed expression of glucose-dependent genes in cones as a marker of glucose transport into the cells and ability to maintain visual pigment-rich OS. Early injection of IVTA at P7 was able to maintain glucose transport and cone OS until at least P65. But, IVTA injection was not able to promote glucose transport or restore cone OS after rods were lost. We then conclude that this positive effect of IVTA on cones is mediated through enhanced survival of mutant rods (Fig. 8*I*). IVTA injection is a commonly used clinical procedure to reduce macular edema in diabetic retinopathy and uveitis (62). It is a quick procedure performed in an office visit, frequently administered every 3 months, and we show that ongoing release of glucocorticoid following injection can protect cone OS in pig RP for a period of at least several months. We suggest that establishment of a CD47 immune checkpoint in conjunction with loss of the cell surface DAMP, CALR, protects mutant rods from PrCR in pig RP and may serve to postpone loss of both rod and cone photoreceptors as disease progresses.

## Experimental procedures

### Experimental design

All methods were approved by the University of Louisville Institutional Animal Care and Use Committee and adhered to the ARVO Statement for Use of Animals in Ophthalmic and Vision Research. WT and P23H *RHO* mutant pigs (7, 70) were compared in the studies. WT and RP pig littermates were followed for retinal apoptosis, expression of cytokines, microglial migration and engulfment of mutant rods, and effects of IVTA and glucose injection at different ages. Ages of animals are shown in figures or provided in legends.

### Randomization

Littermates of different ages were divided into WT and RP groups for experiments. Where indicated, RP littermates were divided into IVTA and sham injection groups. We did not detect differences in female versus male pigs in measurements described above, thus males and females were randomly included into the study groups.

### IVTA injection

Pig were maintained on a 6 AM (on) to 6 PM (off) light cycle and were fed standard chow with no additives. The pigs were sedated with Telzol (2.0–8.8 mg/kg) and intubated, and they were further sedated by intubated anesthesia with 1.5–2% isoflurane mixed with oxygen. 5% Betadine was placed into the eye. After anesthesia and sterile preparation, 0.1 ml of 40 mg/ml (400 ug) triamcinolone was withdrawn into a 1 CC TB syringe. After insertion of an eyelid speculum, triamcinolone was injected 2.5 mm posterior to the limbus in the superior quadrant.

### Statistics

Different investigators performed IVTA injections and histological analysis of retinas. Based on standard deviations derived from our previous extensive studies of rod and cone number changes in WT and RP pigs, we calculated three samples that would be sufficient to detect a 30% change with a confidence of 0.95 in these measurements. We also measured apoptotic cells, PrCR, and real-time PCR for cytokine mRNAs. No previous data was available in the pigs to predict sample numbers for these measurements. However, our results demonstrate that sample numbers for these measurements were sufficient to demonstrate significant changes following IVTA treatment. Each experiment was repeated at least three times. The number of animals and eyes evaluated is shown in the figure legends. Significance was calculated by Student's *t*-test. Error bars in figures show standard deviations.

### Retinal sectioning and immunostaining

Antibodies are described in Table S1. RP and wild control pig eyes at embryos at E65, E85, E105, and postnatal day(P)1, P3, P14, P30, P60, and P90 were harvested and processed for histology and immunohistochemistry. Pigs were euthanized by ear vein injection of beuthanasia (a mixture of sodium pentobarbital and sodium phenytoin, 0.1 ml/lb) through an ear vein catheter after sedation with Ketamine/Dexmedetomidine/Atropine. Embryos were delivered by caesarian section from the euthanized pregnant sows. Both eyes from at least three pigs were used for each time point. Eyes were enucleated and immediately immersed in CO2-independent media on ice. Anterior segment of eyeball was removed and the eye cup for cryosection was fixed in 4% (wt/vol) paraformaldehyde in 0.1 M phosphate buffer for 20 min followed by three washes with 0.1 M phosphate buffer. Tissues were then cryoprotected through 30% sucrose for overnight. Each retina was bisected along the horizontal plane through the dorsal margin of the optic disc and vertically cut through the optic disc. Each of the four pieces was notched on its dorsal edge to preserve orientation.

Retinas were embedded with polyvinyl alcohol, polyethylene-glycol-based optimal cutting temperature cutting reagent (OCT):20% sucrose (2:1). Serial sectioning was performed at 12 μm on a cryostat and tissues were mounted on Super-Frost glass slides. Retinas for paraffin sections were fixed with 10% formalin for 48 h. The retinas were then imbedded with 3% agar gel in 5% formalin and reoriented transversely, then dehydrated in 70% ethanol for paraffin embedding. The tissues were cut at 5 μm, and H&E staining were performed every fifth slide.

Frozen sections were dried at 37 °C for 20 min followed by a rinse through phosphate buffered saline (PBS) for 5 min. The samples were then blocked with 2% bovine serum albumin (BSA), 5% serum, and 0.1% Triton X-100 at 25 °C for 1 h, then incubated with primary antibody at 4 °C overnight. After primary antibodies had been removed and the samples washed, secondary antibodies were applied for 1 h at 25 °C. Photos of immunostained retinal sections were taken with a Zeiss confocal fluorescence microscope, using a 20×, 40×, and 60× magnification, and images were adjusted for contrast and brightness with Adobe Photoshop v9.0.2. As a negative control, no immunostaining was evident in the absence of primary antibodies.

### Counting photoreceptor nuclei and cone OS

Rod and cone nuclei in the ONL were distinguished by nuclear morphology, as in Figure 1*B*. Cone OS were identified by immunostaining for cone opsin (Fig. S2). The diameter of the pig retina is ∼22 mm. Sections were analyzed approximately every 0.5 mm for rod and cone number and cone OS, as displayed in Figure 1*C*. Cell number and cone OS number were counted in three views of ∼800 μM length for each section.

### TUNEL staining for apoptosisrkapoptosis

To identify apoptotic photoreceptor cells, we used a commercially available fluorescent TUNEL kit (DeadEnd Fluorometric TUNEL System, G3250; Promega, USA) according to the manufacturer's instructions. The cell nuclei were counterstained with DAPI. TUNEL-positive rods were counted in different retinal regions described in Figures 1 and S3 and under “counting photoreceptor nuclei and cone OS” above.

### Quantification of PrCR

DAPI staining was used to identify cell bodies in the ONL in the indicated regions of the retina in Figure 4. DAPI+ cell bodies encircled by IBA1+ myeloid cells were designated as cells undergoing PrCR, and the percentage of these cells was calculated in three views corresponding to ∼800 μM in length for each section. At least three eyes were evaluated each retinal region. Sections as in Figure 1*C* were immunostained and counted in Figure 4.

### RNA isolation and real-time PCR

Information regarding PCR primers is shown in Table S1. RNA isolation and real-time PCR were described previously (7). Results were normalized to both beta-Actin and GAPDH mRNA levels with similar results.

## Data availability

All data are shown in the manuscript.

## Conflicts of interest

The authors declare that they have no conflicts of interest with the contents of this article.
