# Supplementary material for: Rod photoreceptor clearance due to misfolded rhodopsin is linked to a DAMP-immune checkpoint switch
Source: J Biol Chem. 2020 Nov 27;296:100102. doi: 10.1074/jbc.RA120.016053 (PMC7949052; doi:10.1074/jbc.RA120.016053)
Supplement: Supplementary file 1 — Figures S1 to S5 and Table S1 [file mmc1.pdf]

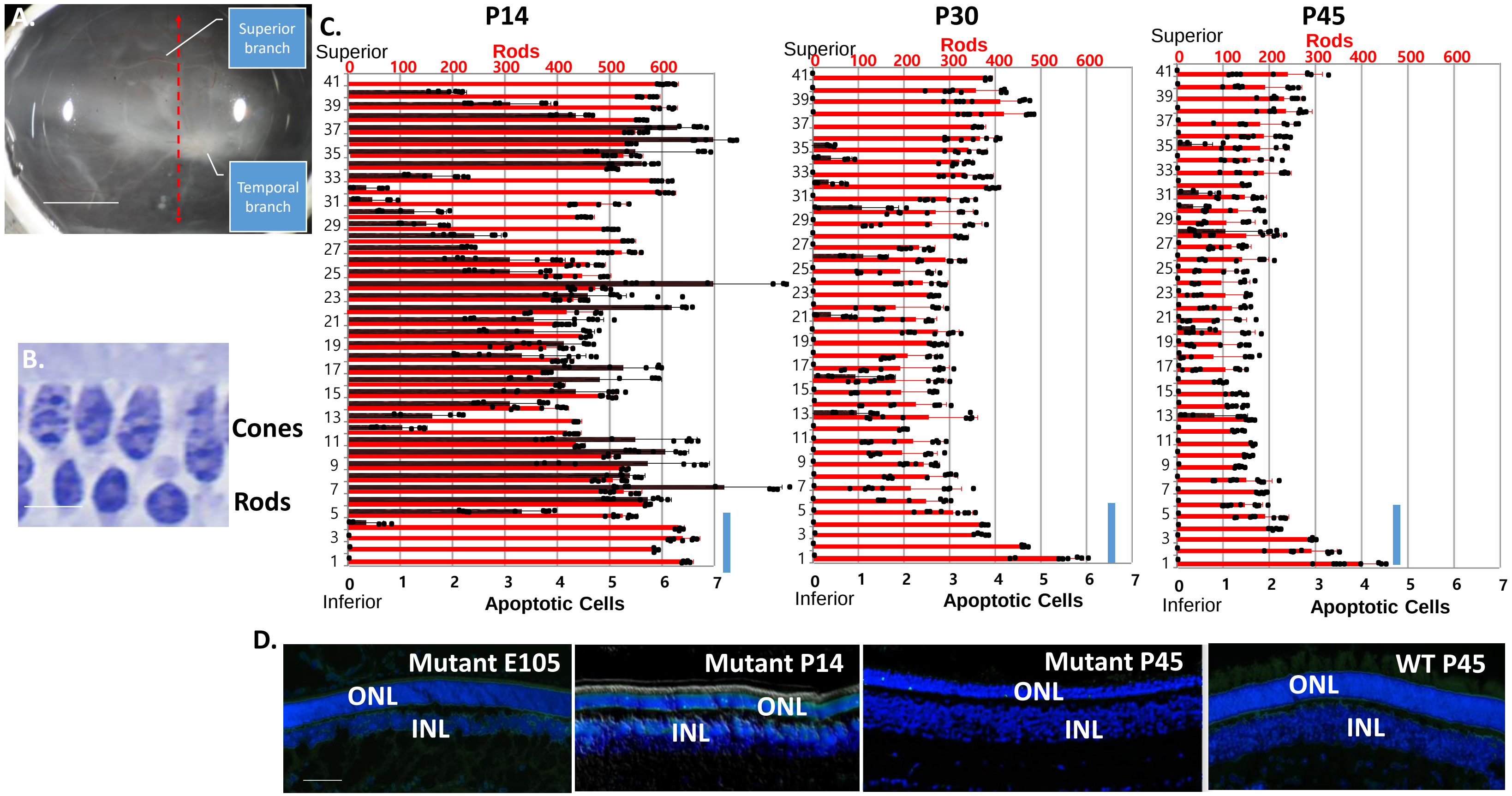

Fig. S1. Regional comparison of rod number to rod apoptosis during pig RP progression. (**A**) Fundus view showing the location of sections for analysis (red arrow). (**B**) H&E staining identifying rod and cone nuclei in the pig retina. Note rods contain compact heterochromatin, whereas cone nuclei have more de-condensed chromatin (Wang et al 2016). Cones are quantified in Fig. S3 and (Wang et al 2016 and 2019). (**C**) The pig retina is ~ 22 mm in diameter. Sections every 0.5 mm were number from inferior to superior position (see panel A). Rods are identified as in panel B. Rods (red bars) and apoptotic cells (black bars) in three high power fields (~800 uM each) were counted for each section in three different retinas. Apoptosis was identified by TUNEL staining (see panel D). Blue bars show an area of rod loss with no detected apoptosis at any of the ages. Also, note loss of rods between P30 and P45 occurs largely in areas where apoptosis was not detected. Bars are averages, and error bars are standard deviations. Further quantification of rod apoptosis during pig RP progression is shown in Fig.S4. (**D**) Representative TUNEL immunostaining at indicated ages. The bar in panel A is 1 mm, panel B 10 um, and panel D is 50 um. “ONL” outer nuclear layer; ”INL”, inner nuclear layer.

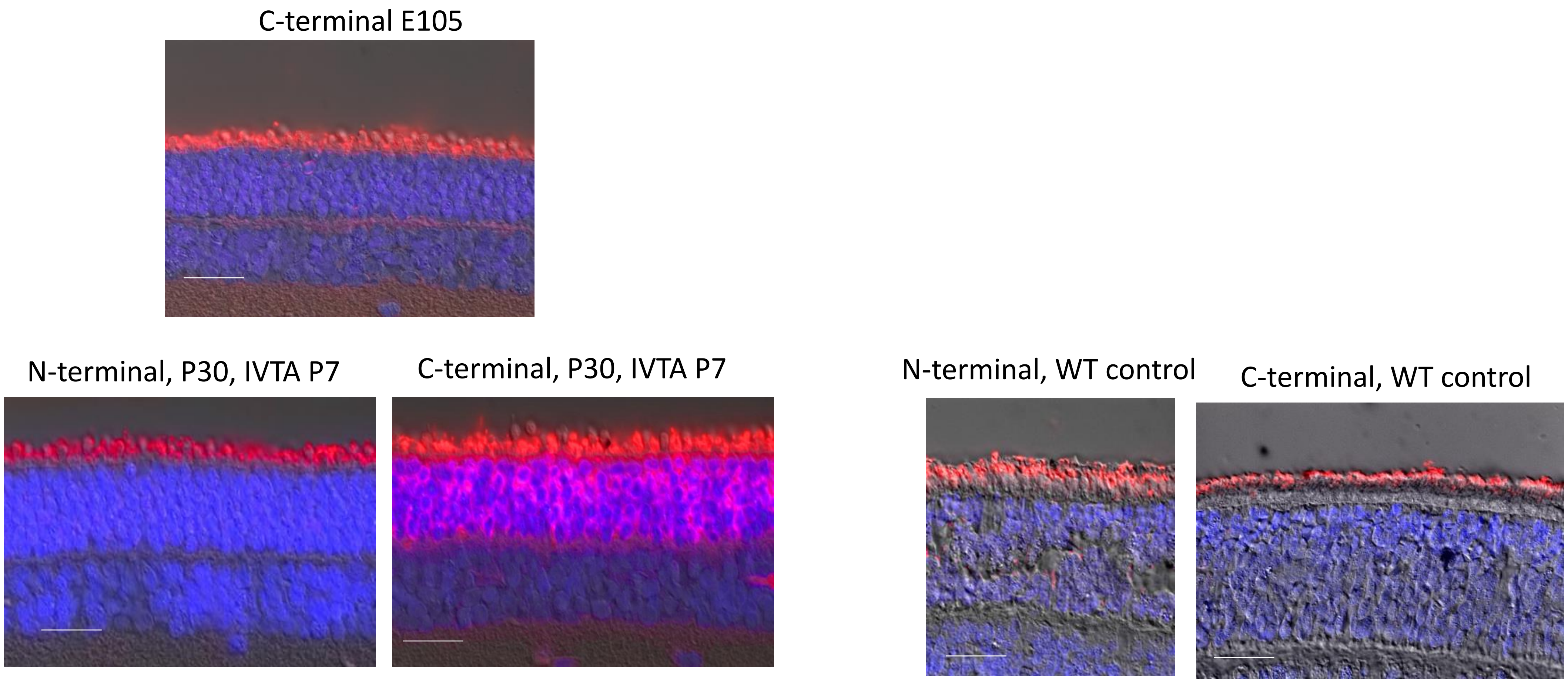

Fig. S2. Immunostaining of WT and RP pig retinas with RHO antibodies to the N- (4D2) and C-terminal (1D4) regions of the protein. Note, that all RHO is in OS prior to birth at E105. Immunostaining with both antibodies shows RHO in rod OS at P30 in WT pigs. The N-terminal antibody shows RHO in OS at P30 after IVTA injection of RP pigs at P7. By contrast, the C-terminal antibody shows RHO in both OS and in the cell body. We conclude that following IVTA injection, RHO diminishes in the ER of IS and is translocated into two pools. The first is OS and the second is the cell body. The N-terminal region appears to be lost from RHO in cell bodies, consistent with degradation of the P23H mutant protein. Representative immunostaining of three retinas is shown. Bars are 50 um. These results support and extend Figs. 5 and 6.

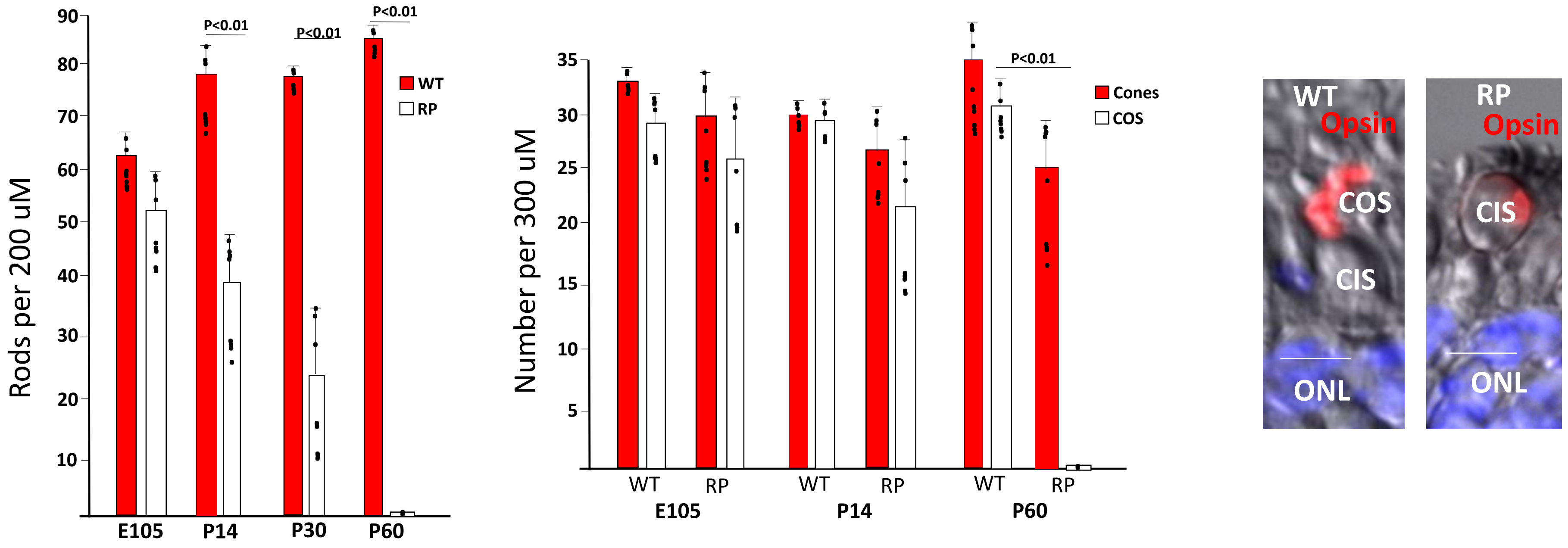

Fig. S3. Quantification of rods and cones as WT and RP pigs age. Results were obtained as described previously (Wang et al 2016) and as in Fig. S1. Three high power images were counted in three different retinas. The region of the retina analyzed is shown in Fig. S1. Rods and cones were identified by H&E staining highlighting differences in heterochromatin density (Fig. S1). Immunostaining for cone opsin was used to quantify cone outer segment (COS). “CIS”, cone inner segment. Bars are 10 um. These results support and extend Figs. S1, 5 and 7.

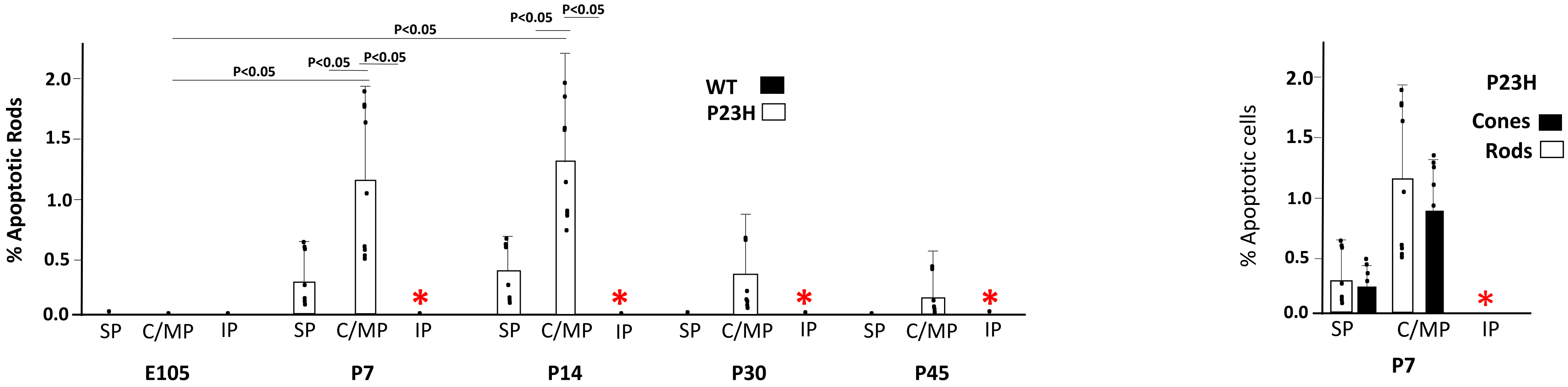

Fig. S4. Left-hand panel. Quantification of TUNEL+ apoptotic rods in the ONL of WT and RP pigs at E105, before birth (gestation is ~110 days) and ending at P45. These results support and extend findings in Fig. S1. The central/mid-peripheral (C/MP) (sections 19-23 in Fig. S1C), superior peripheral (SP) (sections 35-39) and inferior peripheral (IF) (sections 3-7) of WT and RP pig retinas are shown. Right-hand panel. TUNEL+ rods and cones were compared at P7 in P23H pigs, as in the left-hand panel. Rods and cones were identified as in Fig. S1.
* indicates no apoptosis detected despite rod loss in this region (see Fig. S1C, blue bar). Bars are averages and error bars are standard deviations. Three high power fields were counted for each section. Results are representative of three different retinas.

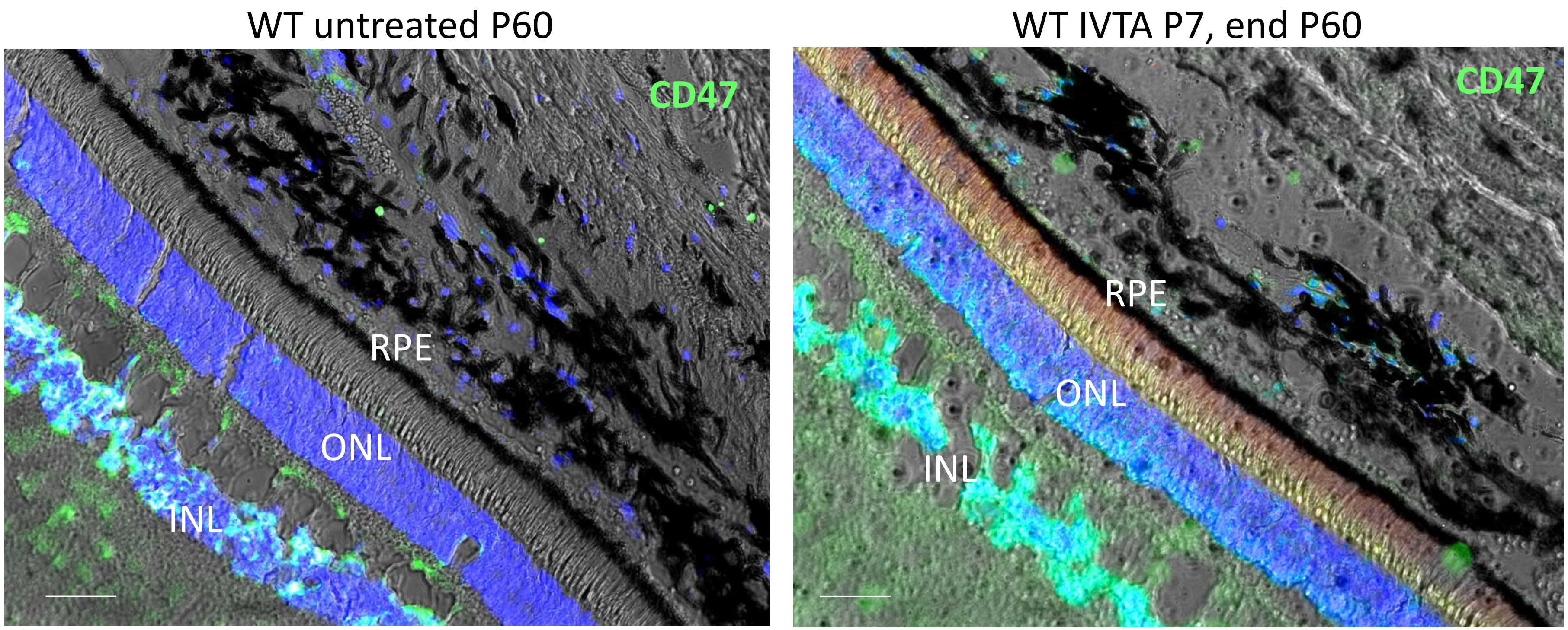

Fig. S5. WT pigs were injected with IVTA or sham control at P7, and retinas were immunostained for CD47 at P60. Note induction of CD47 primarily on rods forming the inner rows of the ONL in the pig. Results are representative of three different retinas. Bars are 100 um. These results support and extend Fig. 6.

Table 1

Real time PCR primers

RNA **Product Size**

Pig	SDF-1	CAGTGTCCCCAGTGTGTCAG	CTCTCAAAGAATCGGCAAGG	160
Pig	TNF-R1	CAGTGTCCCCAGTGTGTCAG	CTCTCAAAGAATCGGCAAGG	201
Pig	TNF-R2	AGGCTGTGTTCATGCCCTAC	GCAGAAACCGAGTTCCAGAG	205
Pig	TRAF-2	ACACGAGCAGGTACGGCTAC	CATCAGGGTCACCTTCTGGT	155
Pig	NF-kappaB p65	TCTGCTTCCAGGTGACAGTG	CCTCGATGTCCTCTTTCTGC	201
Pig	MCP1	CCTTCTGTGGCCTGCTGCTCA	GTCAGCACAGATCTCCTTG	215

Antibodies

1. SDF-1

   Abcam ab9797 1:1000 (Chade et al., 2010)

2. CCL2

   Abcam cat#ab7814

   mybiosource. Catalog#MBS221722.(Lin et al., 2009)

   MCP-1 (clone C4) (Zymed Laboratory). Cross reactive to pig .(Miyata et al., 2000)

   rabbit anti-MCP-1 (1:50, Biovision) (Lin et al., 2009)

3. TNF-α

   TNFa Santa Cruz. Catalog# sc-8301

   rabbit anti-TNFa polyclonal antibody (1:1000 dilution with PBS-BSA; Genzyme, Cambridge, MA) (Nakayama et al., 2003)

   Mouse monoclonal anti-pig TNF-α clone 6E10 (Endogen Inc. Woburn MA) (Trebichavsky et al., 2002)

4. RHO

   mouse anti RHO (Millipore, 1:300)

5. Cone opsin

   chicken anti-JH492 and JH 455 (gifts from Jeremy Nathans, Johns Hopkins Medicine, Baltimore, MD,

1:5000)

6. P-TrkB Abcan (Y816) ab75173. Rabbit. 1:100.
7. P-GR (ser211) cell signaling 4161 Rabbit. (1:100).
8. CALR (1:100) Millipore, 06-661
9. CD47 Abcam ab175388 Rabbit. (1:200)
10. CD73 R&D systems. AF4488 (1:00)
11. SIRPa (1:200) BioLegend, 144013

Bound antibodies were visualized with either Alexa fluor 488- (Invitrogen, 1:500) or Alexa Fluor 568- (Invitrogen, 1:500) conjugated secondary antibodies as described (Wang et al 2016; Wang et al 2019).
